# Diversity and extracellular enzyme activities of heterotrophic bacteria from sediments of the Central Indian Ocean Basin

**DOI:** 10.1038/s41598-019-45792-x

**Published:** 2019-06-28

**Authors:** Vijayshree S. Gawas, Mamatha S. Shivaramu, Samir R. Damare, Devagudi Pujitha, Ram Murti Meena, Belle Damodara Shenoy

**Affiliations:** 1 Biological Oceanography Division, CSIR-National Institute of Oceanography, Dona Paula, 403004 Goa India; 2 CSIR-National Institute of Oceanography Regional Centre, 176, Lawson’s Bay Colony, Visakhapatnam, 530017 Andhra Pradesh India; 3 Department of Bioinformatics, Karunya University, Coimbatore, 611114 Tamil Nadu India

**Keywords:** Marine microbiology, Biodiversity, Environmental impact

## Abstract

Sedimentary bacteria play a role in polymetallic nodule formation and growth. There are, however, limited reports on bacterial diversity in nodule-rich areas of the Central Indian Ocean Basin (CIOB). In this study, bacterial abundance in thirteen sediment cores collected from the CIOB was enumerated, followed by phylogenetic characterisation and, screening of select heterotrophic bacteria for extracellular enzyme activities. Total bacterial counts (TBC) were in the order of 10^7^ cells g^−1^; there was a significant difference (p > 0.05) among the cores but not within the sub-sections of the cores. The retrievable heterotrophic counts ranged from non-detectable to 5.33 × 10^5^ g^−1^; the heterotrophic bacteria clustered within the phyla Firmicutes, Proteobacteria and Actinobacteria. *Bacillus* was the most abundant genus. The extracellular enzyme activities were in the order: amylase > lipase > protease > phosphatase > Dnase > urease. Major findings are compared with previous studies from the CIOB and other areas.

## Introduction

**Figure 1:**
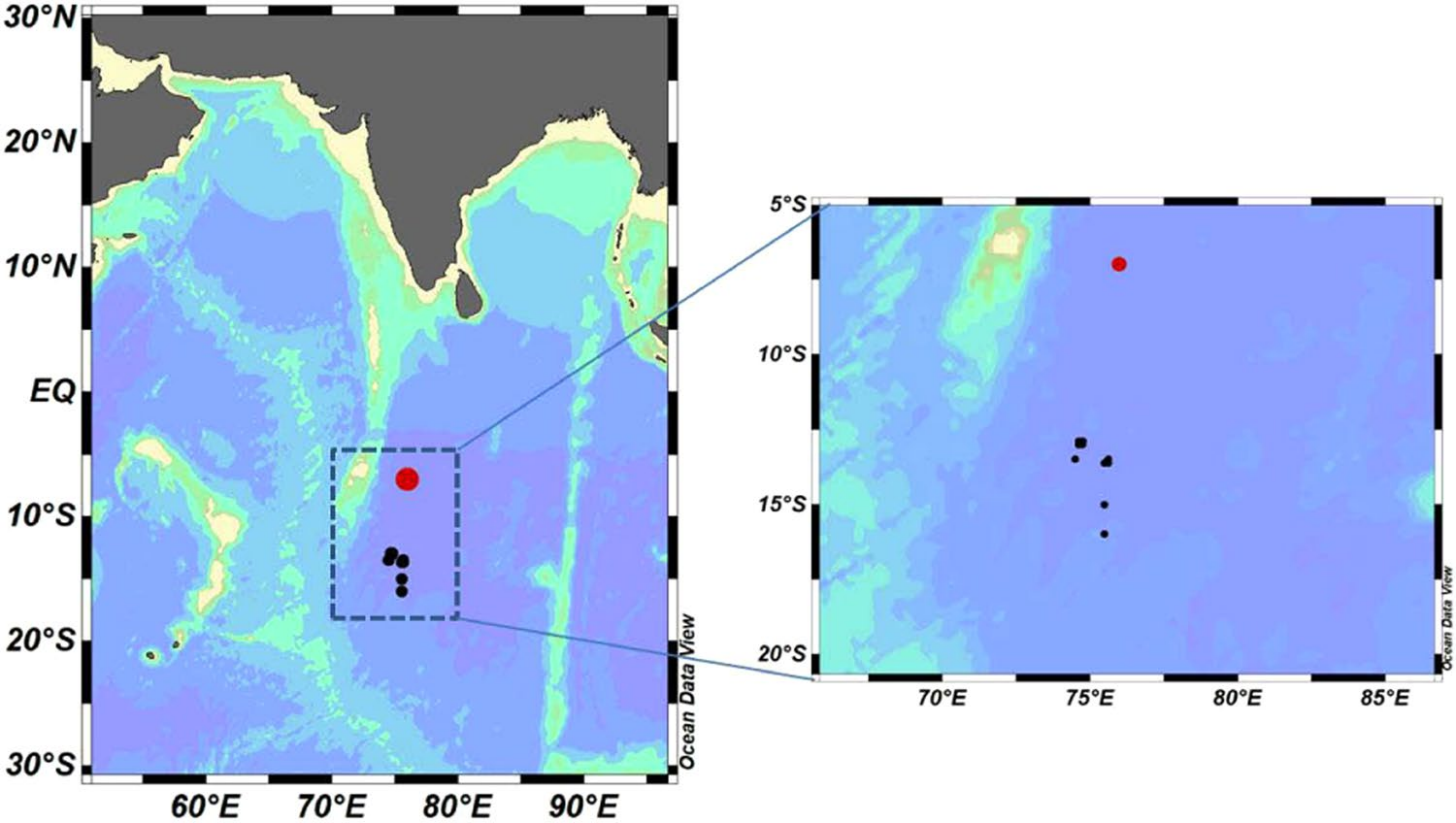
Map showing sampling locations in the Central Indian Ocean Basin. The map was prepared using the tool Ocean Data View^40^ (version 4.7.10), following the attribution guidelines (Schlitzer, R., Ocean Data View, odv.awi.de, 2019).

Polymetallic nodules (PMNs) are found on the seabed and believed to replace the land-based deposits as sources of metals in future^1^. PMNs are majorly composed of concentric layers of iron and manganese oxides around a core, and they are abundantly found at 4000–6000 m water-depth in major oceans^2^. PMNs are rich in metals such as manganese (Mn), nickel (Ni), copper (Cu), cobalt (Co) etc. Regions in the Pacific and Indian Oceans are under consideration for industrial-level mining of PMNs^1^. India is presently conducting environmental impact assessment (EIA) studies in the CIOB (0°–25°S and 70°–90°E)^3^ to evaluate the possible impact of mining of PMNs on the deep-sea environment (Fig. 1)^1^. PMNs are formed abiotically, involving hydrogenetic and diagenetic processes, on the seabed. It is hypothesised that microbial activities, especially of sedimentary bacteria, may contribute towards precipitation of metal hydroxides and play a role in formation and growth of the nodules^4^. These bacteria possibly adsorb metals, including Mn, Fe, Co, Cu and Ni from the deep-sea water and enrich the nodules^5,6^. Sedimentary bacteria are already known to play crucial roles in nutrient cycling in the deep-sea environment^7^, with the help of extracellular enzymes^8^.

Chandramohan *et al*.^9^, in one of the earliest studies from the Indian Ocean (02°N, 58°E), characterised bacteria present in nodules and associated sediments. They reported the presence of both Mn(II)-oxidising and MnO_2_-reducing psychrotrophic bacteria in the nodules. Bacterial taxa *Arthrobacter, Bacillus*, “coryneforms”, *Enterobacteriaceae, Micrococcus*, *Staphylococcus* and *Vibrio* were identified from the nodules and associated sediments. Later, Loka Bharathi & Nair^10^ reported *Acinetobacter*, “coryneforms”*, Enterobacter, Micrococcus, Moraxella, Pseudomonas* and *Staphylococcus* from the sediments of the CIOB. Naik *et al*.^11^ isolated and identified 17 bacterial genera from the sediment samples of the southern and northern regions of the CIOB (Supplementary Table S1).

There are few reports on gene sequence-based identification and taxonomy of culturable bacteria from the CIOB, though there are reports on metagenomic studies [e.g. Das *et al*.^12^, Sujith *et al*.^13^ and Wang *et al*.^14^]. Das *et al*.^12^ performed 454 pyrosequencing of bacterial communities of red clays of the CIOB (16°S, 75.5°E) and reported 14 phyla, 25 classes, 38 orders, 52 families and 62 genera. Sujith *et al*.^13^ studied bacteria from sea-mount samples from the CIOB (03°S, 83°E) and reported the dominance of Proteobacteria and bacterial genera *Halomonas* and *Thiomicrospira*. Recently, Wang *et al*.^14^ characterised the microbial communities in a gravity core sediment sample from rare earth element (REY)-rich mud of the Indian Ocean (22°S, 81°E) using Illumina high-throughput sequencing of 16S rRNA genes and revealed the dominance of Proteobacteria, followed by Firmicutes and Actinobacteria (Supplementary Table S1).

During a cruise (SSD-013) to collect baseline geochemical, biochemical and microbiological data from the First Generation Mining site and the Preservation Reference Zone (PRZ) site, we aimed to enumerate down-core variation of bacterial abundance in the deep sub-seafloor sediments of the CIOB. This was followed by 16S rRNA gene-based phylogenetic characterisation and screening of heterotrophic bacterial cultures for extracellular enzyme activities, including amylase, protease, lipase, DNAse, urease, alkaline phosphatase and nitrate reduction.

## Results

### Abundance of sedimentary bacteria

**Figure 2:**
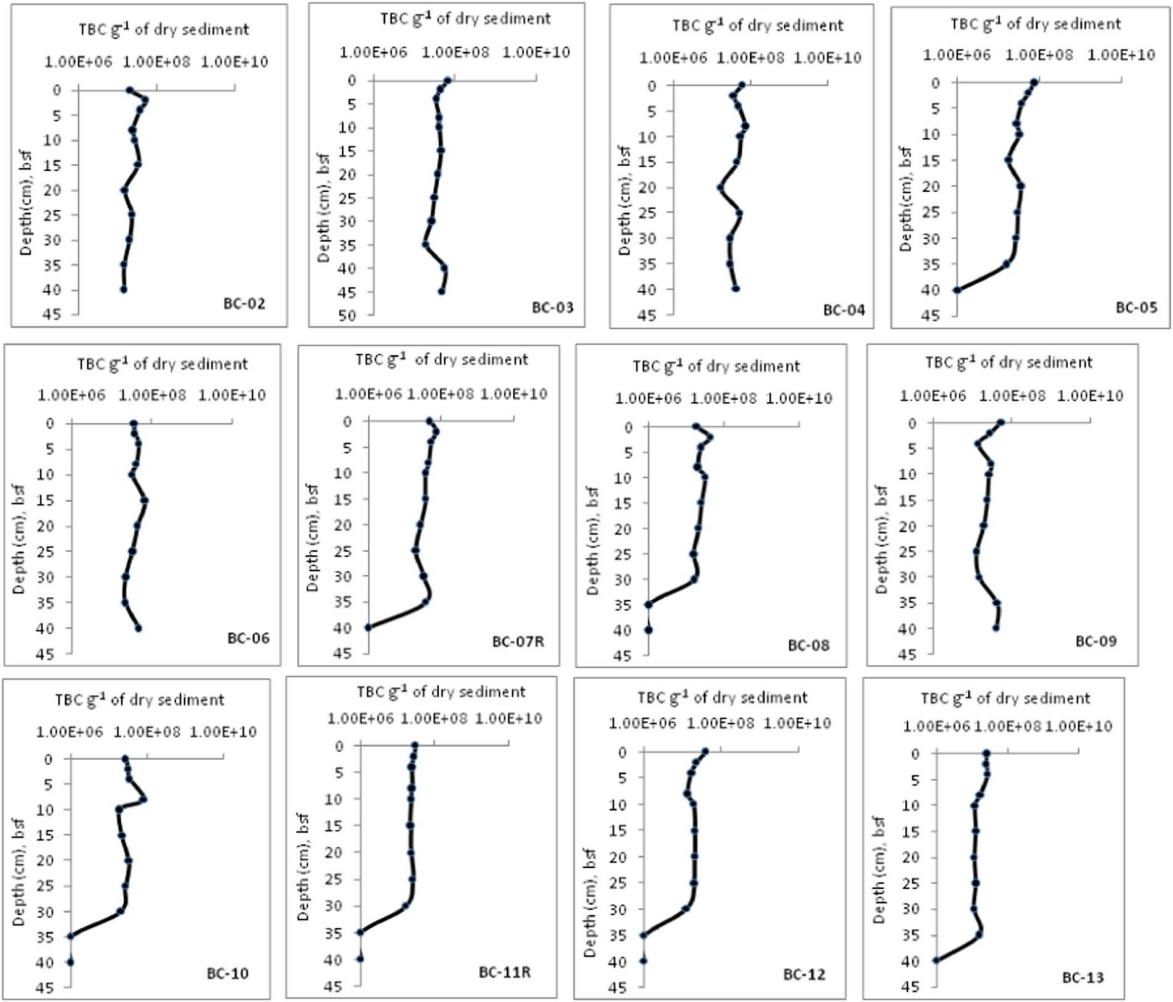
Down-core variation in the total bacterial counts (TBC) in the CIOB sediments.

The water depth at the sampling locations ranged from 4856–5297 m. Down-core variation of total bacterial counts (TBC) in the 13 sediment box-cores is presented in Fig. 2. The TBC were in the order of 10^7^ cells g^−1^ of dry sediment and higher at 0–4 cm bsf. Highest TBC of 8.12 × 10^7^ cells g^−1^ of dry sediment were observed in the BC-10, subsection 6–8 cm bsf. Conversely, lowest TBC of 1.10 × 10^7^ cells g^−1^ of dry sediment were observed in the BC-13, subsection 25–30 cm bsf. Two way ANOVA analysis showed that there is a significant difference (p < 0.05) in the TBC between the sediment cores. However, no significant difference in the TBC was observed within the core sub-sections.

**Figure 3:**
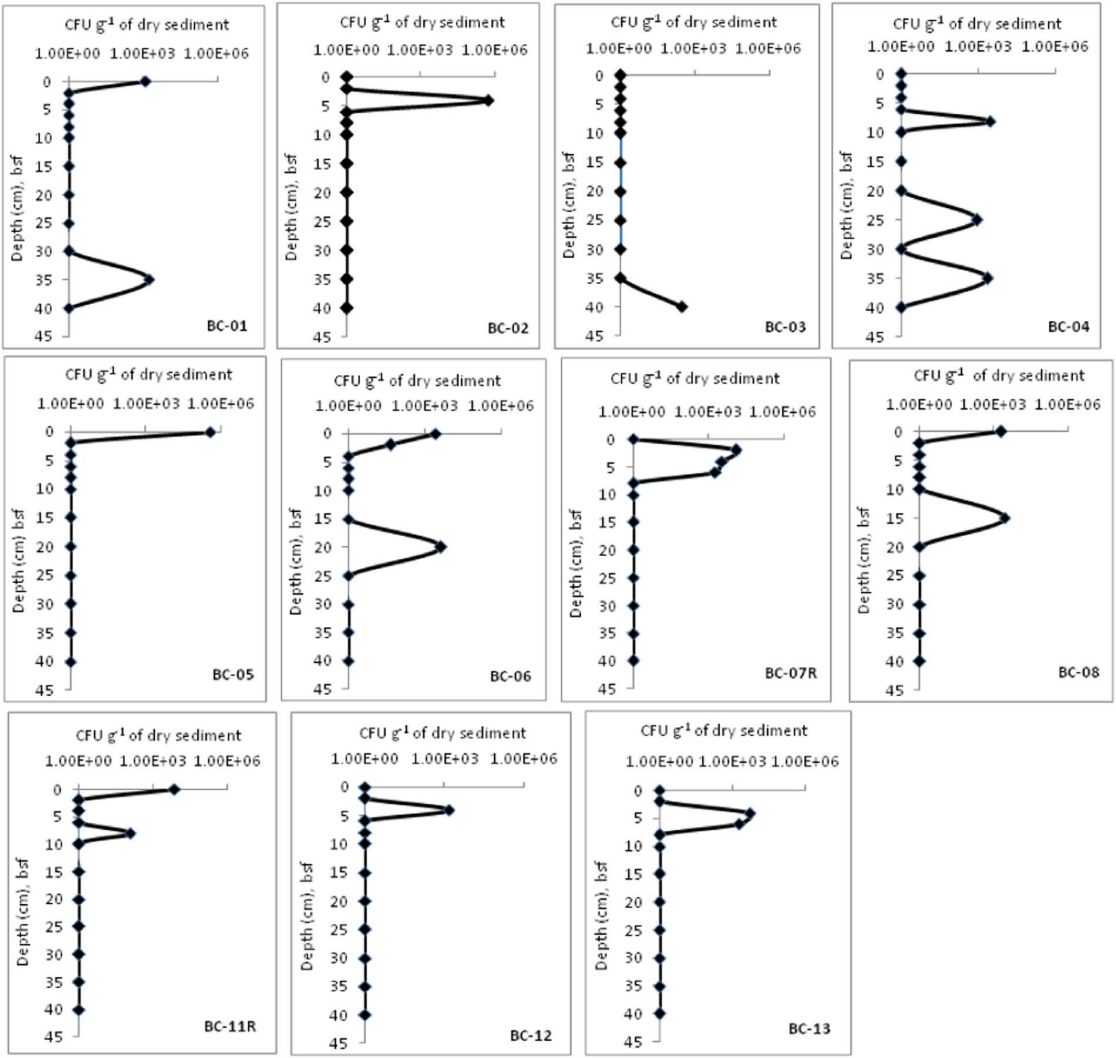
Down-core variation in the heterotrophic bacterial counts (CFUs) in the CIOB sediments.

The retrievable counts of heterotrophic bacteria (colony forming units, CFUs) performed in duplicates and averaged later are presented in Fig. 3. The CFUs ranged from non-detectable to 5.33 × 10^5^ g^−1^ of dry sediment. Down-core variation in the CFUs was higher at 4–6 cm bsf. The highest CFUs were recovered from the BC-02, sub-section 4–6 cm bsf. Higher CFUs were recovered from the upper 10 cm, compared to the deeper sub-sections of the cores (Fig. 3). The heterotrophic bacteria were not detectable in the cores BC-09 and BC-10, though the TBC from the cores were in the order of 10^7^ g^−1^ of dry sediment.

### Phylogenetic characterisation of heterotrophic bacteria

**Figure 4:**
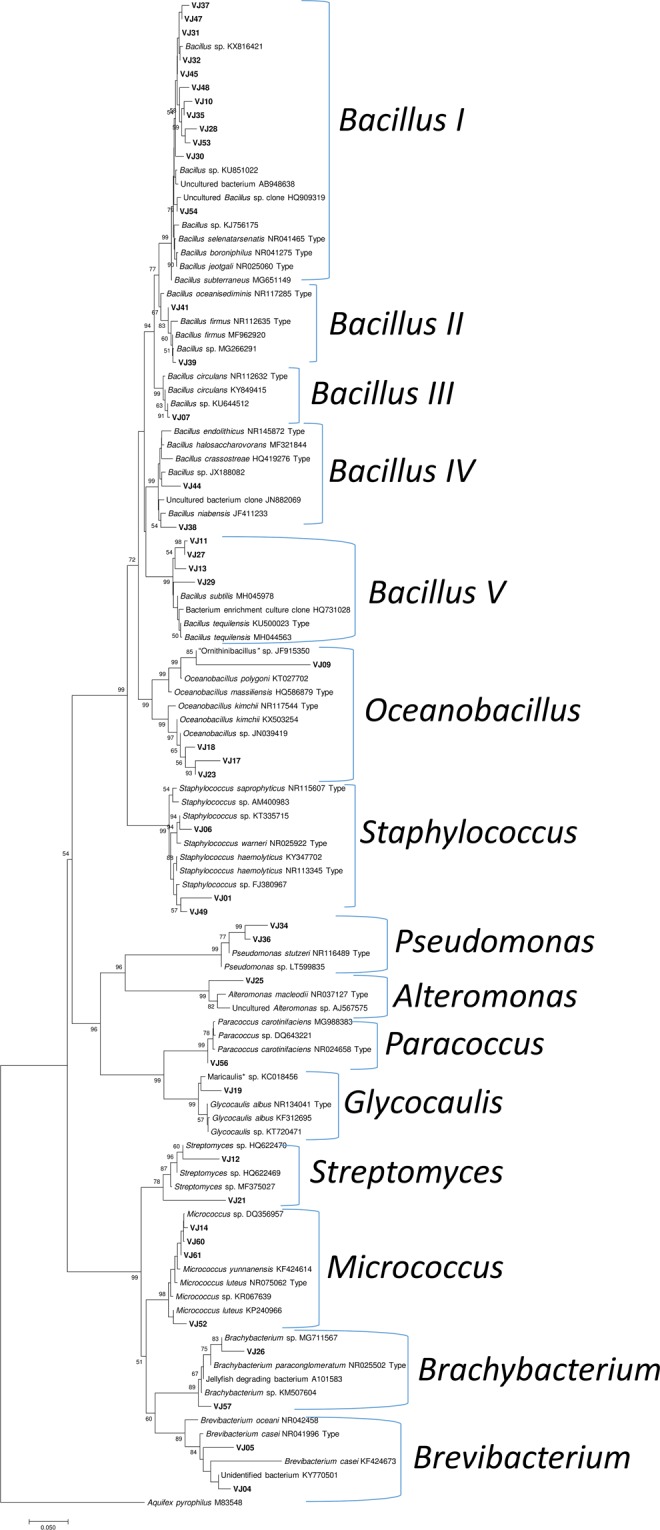
Phylogenetic characterization of select heterotrophic bacteria based on 16S rRNA gene sequence analysis in MEGA.

In total, 62 heterotrophic bacteria were isolated. Only 43 bacterial cultures were included in the phylogenetic analysis due to poor viability of the 19 cultures. Phylogenetic relationships of the bacteria, inferred based on 16S rRNA gene sequence-data analysis, are presented in Fig. 4. The system output details retrieved from MEGA 7^15^ about the tree construction are in Supplementary Document. In the NJ tree (Fig. 4), 43 newly generated gene-sequences clustered within three bacterial phyla, Actinobacteria, Firmicutes and Proteobacteria (subphyla α-Proteobacteria and γ-Proteobacteria). The phylogenetic analysis recovered 15 bacterial clades, including *Bacillus* clades I-V, *Oceanobacillus*, *Staphylococcus* (Firmicutes), *Alteromonas*, *Glycocaulis*, *Paracoccus*, *Pseudomonas* (Proteobacteria), *Brachybacterium*, *Brevibacterium*, *Micrococcus* and *Streptomyces* (Actinobacteria) (Fig. 4). Location-wise and subsection-wise groupings of the identified clades are presented in Supplementary Tables S2 and S3, respectively.

### Screening for extracellular enzyme activities

The enzyme activities of the 43 bacterial cultures followed the order: amylase > lipase > protease > phosphatase > Dnase > urease. Nine cultures were positive for nitrate reduction. Nine *Bacillus* cultures isolated from 4–6 cm subsection bsf were positive for most of the enzyme activities (Table 1). *Streptomyces* sp. VJ21 was positive for all the extracellular enzyme activities and nitrate reduction. *Staphylococcus* sp. VJ01 was positive for only nitrate reduction. *Alteromonas* sp. VJ25 was negative for all the enzyme activities and nitrate reduction.

**Table 1:** List of bacterial cultures included in the phylogenetic analysis, their extracellular enzyme activities and nitrate reduction.

Sl. no.	Clade name	Isolate no.	Station no.	Core depth (cm)	Amylase	Protease	Lipase	Dnase	Urease	Alkaline phosphatase	Nitrate reduction	Genbank no.
1	*Alteromonas*	VJ25	BC-06	35–40	−	−	−	−	−	−	−	MH605399
2	*Bacillus* clade I	VJ10	BC-02	4–6	+	−	+	+	−	−	−	MH605389
3	*Bacillus* clade I	VJ28	BC-07R	2–4	+	−	−	−	−	−	−	MH605402
4	*Bacillus* clade I	VJ30	BC-07R	4–6	+	+	−	−	−	−	−	MH605404
5	*Bacillus* clade I	VJ31	BC-07R	6–8	+	−	−	−	−	−	−	MH605405
6	*Bacillus* clade I	VJ32	BC-07R	6–8	+	+	−	−	−	−	−	MH605406
7	*Bacillus* clade I	VJ35	BC-07R	6–8	−	−	−	−	−	−	−	MH605408
8	*Bacillus* clade I	VJ37	BC-07R	6–8	+	+	+	−	−	−	−	MH605410
9	*Bacillus* clade I	VJ45	BC-08R	15–20	−	−	−	−	−	−	−	MH605415
10	*Bacillus* clade I	VJ48	BC-10	30–35	+	−	+	−	−	−	−	MH605417
11	*Bacillus* clade I	VJ47	BC-10	35–40	+	−	+	−	−	−	−	MH605416
12	*Bacillus* clade I	VJ53	BC-12	4–6	+	−	+	−	−	−	+	MH605420
13	*Bacillus* clade I	VJ54	BC-12	4–6	+	−	+	−	−	+	−	MH605421
14	*Bacillus* clade II	VJ41	BC-08R	2–4	−	−	−	−	−	−	−	MH605413
15	*Bacillus* clade II	VJ39	BC-08R	4–6	−	+	−	+	−	+	−	MH605412
16	*Bacillus* clade III	VJ07	BC-02	30–35	+	−	−	+	−	+	+	MH605387
17	*Bacillus* clade IV	VJ38	BC-08R	4–6	−	+	−	−	−	+	−	MH605411
18	*Bacillus* clade IV	VJ44	BC-08R	8–10	−	−	−	−	−	−	−	MH605414
19	*Bacillus* clade V	VJ11	BC-03	40–45	+	+	+	+	−	+	+	MH605390
20	*Bacillus* clade V	VJ13	BC-04	8–10	+	+	+	+	−	+	+	MH605392
21	*Bacillus* clade V	VJ27	BC-06	20–25	+	+	+	+	−	−	+	MH605401
22	*Bacillus* clade V	VJ29	BC-07R	2–4	+	+	+	+	−	+	−	MH605403
23	*Brachybacterium*	VJ26	BC-06	20–25	+	+	−	−	−	+	−	MH605400
24	*Brachybacterium*	VJ57	BC-13	4–6	+	+	+	−	−	+	−	MH605423
25	*Brevibacterium*	VJ04	BC-01	0–2	−	−	−	−	−	+	−	MH605384
26	*Brevibacterium*	VJ05	BC-01	0–2	−	−	−	−	−	+	−	MH605385
27	*Glycocaulis*	VJ19	BC-05	0–2	−	−	−	−	−	+	−	MH605396
28	*Micrococcus*	VJ14	BC-04	25–30	−	−	−	−	−	−	−	MH605393
29	*Micrococcus*	VJ52	BC-11R	0–2	+	+	+	−	−	−	−	MH605419
30	*Micrococcus*	VJ61	BC-13	4–6	+	−	+	−	−	−	−	MH605425
31	*Micrococcus*	VJ60	BC-13	0–2	+	+	+	−	−	−	−	MH605424
32	*Oceanobacillus*	VJ09	BC-02	4–6	+	+	+	+	−	−	−	MH605388
33	*Oceanobacillus*	VJ17	BC-04	8–10	−	+	+	−	−	−	−	MH605394
34	*Oceanobacillus*	VJ18	BC-04	35–40	−	+	+	−	−	−	−	MH605395
35	*Oceanobacillus*	VJ23	BC-06	2–4	−	−	+	−	−	−	−	MH605398
36	*Paracoccus*	VJ56	BC-13	6–8	+	−	−	−	−	−	−	MH605422
37	*Pseudomonas*	VJ34	BC-07R	6–8	+	−	−	−	−	−	+	MH605407
38	*Pseudomonas*	VJ36	BC-07R	6–8	+	−	+	−	−	−	+	MH605409
39	*Staphylococcus*	VJ01	BC-01	0–2	−	−	−	−	−	−	+	MH605383
40	*Staphylococcus*	VJ06	BC-01	35–40	−	−	−	−	−	−	−	MH605386
41	*Staphylococcus*	VJ49	BC-11R	2–4	−	−	−	−	−	−	−	MH605418
42	*Streptomyces*	VJ12	BC-04	8–10	−	−	−	−	−	++	−	MH605391
43	*Streptomyces*	VJ21	BC-05	0–2	+	+	+	+	+	++	+	MH605397

## Discussion

This is the first study reporting 16S rRNA gene-based phylogenetic characterisation of heterotrophic bacteria from sub-seafloor sediments of PMN-rich areas in the CIOB. The forty-three heterotrophic bacteria clustered within three phyla and 11 known genera (Table 1). *Bacillus* species were majorly isolated, which formed 5 distinct clades*. Bacillus* is widely-reported from the CIOB and other parts of the Indian Ocean^9–13,16,17^, especially in PMNs and associated sediments. Further, four bacteria grouped within *Oceanobacillus*. *Oceanobacillus* is a new record from the sediments of the CIOB. It appears that culturable bacilli are abundant in the PMN-rich sediments of the CIOB. Similar patterns of dominance by Firmicutes, including bacilli, were reported by Sujith *et al*.^13^, Wang *et al*.^14^, and Khandeparker *et al*.^16^ from the Indian Ocean. Bacilli are known to survive and grow under physicochemical extremities and with high metabolic versatility. This possibly can explain the abundance of culturable bacilli in the CIOB sediments.

To the best of our knowledge, *Brachybacterium*, a member of the phylum Actinobacteria, has not been reported from the CIOB (Supplementary Table S1), even though they are known from deep-sea sediments of Southern Ocean^18^ and Pacific Ocean deep-sea Mn nodule sediment^19^. This genus is reported for the first time from the CIOB sediment samples. Interestingly, *Brachybacterium* sp. isolated from the nodules in the Pacific Ocean^19^ reportedly showed high Mn(II) resistance and Mn(II)-oxidizing/removing abilities. *Brevibacterium*, a member of “coryneforms” bacteria reported in this study, might have been also isolated from the CIOB sediments and classified under “coryneforms” by Loka Bharathi & Nair^10^. Chandramohan *et al*.^9^ and Sujith *et al*.^17^ reported “coryneforms” and *Brevibacterium* members from the nodules and basal fragments collected from the non-CIOB regions of the Indian Ocean, respectively (Supplementary Table S1).

*Glycocaulis*, to the best of our knowledge, has not been previously reported from the CIOB (Supplementary Table S1). The genus was originally described from hot water plume of a deep-sea hydrothermal vent from Canada and it reportedly lacks phospholipids^20^. Role of bacterial phospholipids in deep-sea ecology is subject to future studies. *Micrococcus* is previously reported from the sediments of the CIOB by Loka Bharathi & Nair^10^ and Naik *et al*.^11^. Chandramohan *et al*.^9^ reported this genus from nodules and associated sediments from a non-CIOB region in the Indian Ocean. *Micrococcus* species are known to have the ability to adapt to elevated pressure^21^, while some species of *Micrococcus* may produce antibiotics^22^.

*Pseudomonas* species have been reported from the CIOB sediments (Supplementary Table S1) and also from a few non-CIOB regions. *Pseudomonas* species are reportedly involved in Mn-oxidation^5,6^, and they possibly adsorb metals such as Co, Cu, and Ni. Interestingly, they may form biofilms which help in the growth of nodules^23^. *Paracoccus* has not been previously reported from the CIOB sediments, though the genus is known from deep sediments of the Arctic Ocean^24^.

*Staphylococcus* has been reported from the CIOB and the non-CIOB regions (Supplementary Table S1). Recently, Wang *et al*.^25^ reported the whole genome sequence of a Mn(II) tolerant *Staphylococcus* sp. isolated from deep-sea sediment in the Clarion-Clipperton Zone in the Pacific ocean, which exhibited efficient Mn(II) oxidation. Further studies are required from the CIOB about molecular mechanisms and pathways involved in Mn(II) oxidation by bacteria such as *Staphylococcus* spp. Bacterial genus *Streptomyces* is reported for the first time from the CIOB sediments. *Streptomyces *is known for the production of secondary metabolites. Recently, Rodríguez *et al*.^26^ reported *Streptomyces cyaneofuscatus* isolated from a Gorgonian coral collected at 1500 m depth in the Avilés submarine Canyon which produced antibiotic Anthracimycin B. Role of bacterial secondary metabolites, including antibiotics, in deep-sea ecology of the CIOB, needs further investigations.

In our study, we observed that the TBC were two orders higher than the CFUs. The TBC were in the order 10^7^ g^−1^ of dry sediment. Similar counts were reported by Naik *et al*.^11^ from the CIOB, while Raghukumar *et al*.^27^ reported TBC two orders higher than our findings. There was no significant difference in the TBC within the core sub-sections, suggesting a homogeneous distribution pattern of total bacterial abundance. Loka Bharathi and Nair^10^ and Raghukumar *et al*.^27^ reported similar homogeneous distribution patterns. Geological studies from the CIOB indicate that sediment characteristics (e.g. porous, mottled and loose)^28^ and disturbance of sediment layers by biological activities such as bioturbation^11^ could be responsible for this kind of homogeneous distribution pattern. ISBA guidelines (2018) recommend the use of molecular tools to quantify bacterial abundance in the proposed PMN-mining areas. Traditional methods, therefore, may be replaced with modern molecular tools in microbial abundance studies, in near future E.g. Cho *et al*.^29^.

The cultivability of heterotrophic bacteria in our study was about 1% of the TBC. Similar results were reported by Nair *et al*.^30^ and Raghukumar *et al*.^27^ from the CIOB and by Chandramohan *et al*.^9^ from a non-CIOB region. Naik *et al*.^11^, however, reported CFU counts one order lesser than the present study. The retrievable counts of heterotrophic bacteria have been used to quantify variations in bacterial abundance under the influence of disturbance in environmental conditions. For example, Loka Bharathi and Nair^10^ observed an increase in retrievable counts from 10^2^ to 10^4^ g^−1^ of dry sediment in response to benthic disturbance in the CIOB. Culture-based CFU studies also result in microbial cultures which could be employed in ecological and biotechnological studies.

The 43 heterotrophic bacterial cultures screened for seven extracellular enzymatic activities showed promising results. Hydrolytic degradation of polymers such as starch, protein and lipids by bacterial cultures is a possible indicator of mineralisation of polymers and regeneration of nutrients in the deep-sea^27^. In this study, 25 bacterial cultures exhibited amylase activity. Chandramohan *et al*.^9^, Raghu kumar *et al*.^27^ and Loka bharathi and Nair^10^ have previously reported amylase activity from the bacterial cultures from the Indian Ocean. Loka Bharathi and Nair^10^ suggested that amylase activity by bacterial cultures from the sediments indicates the availability of free sugar in the CIOB sediments. Lipase activity was observed in 20 bacterial cultures (Table 1), suggesting *in situ* lipolytic activity of the bacteria^9,10,27^. Seventeen cultures were positive for protease activity, and possibly these bacteria are involved in break-down of complex proteins present in the sediments into simpler peptides and amino acids. Fourteen cultures were positive for phosphatase activity. Recently, Biche *et al*.^31^ reported phosphatase activities of the sediment samples as an indication of available phosphate in the CIOB sediments. The bacterial enzymatic activities of phosphatases and nitrate reductases in the present study indicate that the sedimentary bacterial communities possibly participate in nutrient recycling. These are preliminary observations revealed in this study, which need to be subjected to further research for confirmation.

## Conclusion

This is the first study reporting 16S rRNA gene-based phylogenetic characterization of heterotrophic bacteria from the sediments of potential PMN-mining areas in the CIOB. As evident from the present study and other studies, PMNs and associated sediments support phylogenetically-diverse bacterial diversity, which is reportedly distinct from the deep water column^32^ (Supplementary Table S1). A comprehensive understanding of microbial diversity present in nodule-rich areas of the CIOB is required to assess the impact of large scale removal of nodules and sediments from the deep sea-bed for industrial mining of the PMNs, as suggested Schulse *et al*.^32^ in the context of Pacific Ocean. Microbial abundance, diversity and functions can vary across the seabed^33^, therefore studies employing culture-dependant and -independent methods are required to improve our understanding of microbial diversity and their role in deep-sea ecology.

## Methods

### Sampling

Sampling was carried out during July–August 2015 with Research Vessel Sindhu Sadhana (SSD-013), as part of ongoing Polymetallic Nodules Program (PMN). The sub-seafloor sediment samples were collected from 13 locations in the CIOB (Fig. 1; Supplementary Table S4), using USNEL-type box-corer of 50 cm × 50 cm × 50 cm dimension.

The box-corer was partitioned into four parts by fixing metallic plates. After siphoning the overlying water, sub-samples were collected using clean 6.3 mm inner diameter plexiglass tubes. On an average, 45 cm bsf long cores were obtained. The cores were sliced at 2 cm intervals up to 10 cm depth and at 5 cm intervals thereafter and extruded aseptically to sterile polythene bags on board. The bags with sediment samples were preserved at 4 °C until further processing.

### Abundance of bacteria in deep sub-seafloor sediment samples

For TBC, approximately 1 g of sediment sample was suspended in 9 ml of sterile seawater and processed as detailed in Das *et al*.^34^. The DAPI method was used to count the cells. Total bacterial counts were expressed as cells g^−1^ of dry sediment^35^.

For retrievable counts of heterotrophic bacteria, each sub-sample was serially diluted to 10^–2^ with filtered sterile seawater as diluent on-board. Zobell marine agar (ZMA, 12.5%) medium (HiMedia, India) prepared with 50% seawater was spread-plated with 100 μl of original and 10^−2^ inocula, in duplicates, and incubated at 4 °C for a month and later transferred to 25 °C for another 15 days. After incubation, the plates were examined for retrievable CFUs and calculated per gram of dry sediment.

Bacterial colonies with apparent unique morphology were selected and purified by quadrant streaking method. All the purified bacterial cultures were maintained on ZMA by sub-culturing after every four months as recommended by Bowman^36^ for further studies. The bacterial cultures were also preserved in 80% glycerol stocks and stored -80 °C for long-term.

### DNA extraction and PCR amplification of 16S rRNA gene

Select bacterial cultures were cultivated in Zobell marine broth (ZMB, 12.5%) for 3–7 days at 25 °C, followed by DNA extraction using ZM Fungal/ Bacterial DNA MiniPrep Kit (Zymo Research, D6005). 16S rRNA gene was amplified by PCR using the primers 27F (5′-AGAGTTTGATCCTGGCTCAG-3′) and 1492R (5′-TACGGYTACCTTGTTACGACTT-3′)^37^. PCR amplification was performed in a reaction volume of 50 μl, including 5 μl of 10X concentrated buffer (with 15 mM MgCl_2_) (Genei, Bangalore, India), 1 μl of 10 mM dNTP mix (Genei), 1 μl each of 20 pm/µl 27F and 1492R primers, 1.5 μl of Taq polymerase (1 unit/ μl) (Chromous Biotech, India), 3 μl 20–50 ng template DNA and 37.5 μl nuclease free water.

PCR conditions used were: initial denaturation of 5 min at 95 °C, followed by 35 cycles of 1 min at 95 °C, 1 min at 50 °C and 2 min at 72 °C, and a final extension of 72 °C for 10 min. Purification of PCR product was carried out using QIAquick PCR purification columns (QIAGEN, Catalogue number 28106), following the manufacturer’s instructions. The PCR amplicons (DNA) were stored at -20 °C.

### Gene sequencing and phylogenetic analysis

The purified PCR products were sequenced using Genetic Analyzer 3130xl (ABI) based on the Big Dye termination v 3.1 (Chain termination) chemistry using the same primer set. For each isolate, a consensus sequence was generated in DNA Dragon (SequentiX, Germany) using both forward and reverse sequences, where possible.

A multiple alignment of 16S rRNA gene sequences, including newly generated sequences from this study and homologous sequences retrieved from NCBI GenBank (Fig. 2), was prepared in MEGA. The 16S rRNA gene sequences generated in this study have been deposited in the NCBI GenBank under accession numbers MH605383–MH605425 (Table 1). Phylogenetic relationships of the bacteria from deep-sea sediments were evaluated in MEGA.

### Screening for extracellular enzyme activities

The 43 bacterial cultures were screened for extracellular hydrolytic enzyme activities, including amylases, proteases, lipases, DNases and ureases on agar medium having 1% substrates, i.e. starch, casein, tween 80, toluidine blue and urea, respectively^27^. Alkaline phosphatase^38^ and Nitrate reductase^39^ were also checked for these cultures. After spot inoculation, the plates were incubated for 4 days at 25 ± 1 °C.

### Statistical analysis

Two-way analysis of variance (ANOVA) was carried out in Statistica ver. 6 (Statsoft) to find out significant variations in bacterial abundance within the depths and between the cores.

## Supplementary information

Supplementary information: Legends and data

Supplementary Table 1
