# Supplementary material for: Diversity and extracellular enzyme activities of heterotrophic bacteria from sediments of the Central Indian Ocean Basin
Source: Sci Rep. 2019 Jun 28;9:9403. doi: 10.1038/s41598-019-45792-x (PMC6599205; doi:10.1038/s41598-019-45792-x)
Supplement: Supplementary file 1 — Supplementary information: Legends and data [file 41598_2019_45792_MOESM1_ESM.pdf]

Diversity and extracellular enzyme activities of heterotrophic bacteria from sediments of the Central Indian Ocean Basin

Authors:

Vijayshree S Gawas[1], Mamatha S Shivaramu[1, *], Samir R Damare[1], Devagudi Pujitha[2, 3], Ram Murti Meena[1], Belle Damodara Shenoy[2, **]

Affiliations:

[1]Biological Oceanography Division, CSIR-National Institute of Oceanography, Dona Paula - 403004, Goa, India

[2]CSIR-National Institute of Oceanography Regional Centre, 176, Lawson's Bay Colony, Visakhapatnam - 530017, Andhra Pradesh, India

[3]Department of Bioinformatics, Karunya University, Coimbatore - 611114, Tamil Nadu, India

Corresponding authors:

*Mamatha S Shivaramu

E-mail: mamatha@nio.org

**Belle Damodara Shenoy

E-mail: belleshenoy@nio.org

Supplementary Table S1: List of bacterial taxa reported from the CIOB and a few non-CIOB regions in the Indian Ocean

Supplementary Table S2: Sampling location-wise delineation of the identified bacterial clades

Supplementary Table S3: Sediment core-depth-wise delineation of the identified bacterial clades

Supplementary Table S4: Sampling data collected for each of the sampling station

Supplementary Document: Output details retrieved from MEGA software for the phylogenetic tree construction

Supplementary Table S2: Sampling location-wise delineation of the identified bacterial clades

Station	Bacterial clades recovered
**BC-01**	*Bacillus* clade I, *Staphylococcus,Brevibacterium*
**BC-02**	*Bacillus* clade I, *Bacillus* clade III, *Oceanobacillus*
**BC-03**	*Bacillus* clade V
**BC-04**	*Bacillus* clade V, *Oceanobacillu, Streptomyces, Micrococcus*
**BC-05**	*Glycocaulis*, *Brevibacterium*
**BC-06**	*Bacillus* clade V, *Oceanobacillus*, *Alteromonas, Brachybacterium*
**BC-07R**	*Bacillus* clade I, *Bacillus* clade V, *Pseudomonas*
**BC-08R**	*Bacillus* clade I, *Bacillus* clade II, *Bacillus* clade IV
**BC-09**	None reported
**BC-10**	*Bacillus* clade I
**BC-11R**	*Staphylococcus*, *Micrococcus*
**BC-12**	*Bacillus* clade I
**BC-13**	*Paracoccus*, *Micrococcus*, *Brachybacterium*

Supplementary Table S3: Sediment core-depth-wise delineation of the identified bacterial clades

**Core depth (cm)**	**Bacterial clades recovered**
**0-2**	*Staphylococcus*,*Glycocaulis*, *Brevibacterium*, *Micrococcus*
**2-4**	*Bacillus* clade I, *Bacillus* clade II, *Bacillus* clade V, *Oceanobacillus*, *Staphylococcus*
**4-6**	*Bacillus* clade I, *Bacillus* clade II, *Bacillus* clade IV, *Oceanobacillus*, *Micrococcus*, *Brachybacterium*
**6-8**	*Bacillus* clade I, *Pseudomonas, Paracoccus*
**8-10**	*Bacillus* clade IV, *Bacillus* clade V, *Oceanobacillus*, *Streptomyces*
**10-15**	None reported
**15-20**	*Bacillus* clade I
**20-25**	*Bacillus* clade V, *Brachybacterium*
**25-30**	*Micrococcus*
**30-35**	*Bacillus* clade I, *Bacillus* clade III
**35-40**	*Bacillus* clade I, *Oceanobacillus, Staphylococcus*, *Alteromonas*
**40-45**	*Bacillus* clade V

Supplementary Table S4: Sampling data collected for each of the sampling station

Sl. no.	Station no.	Touch positions		Water depth (m)	Core Depth (cm)
		**Lat (S)**	**Long (E)**		
**1**	BC-01	07° 00' 002"	75° 59' 994"	5297	0-40
**2**	BC-02	12° 52' 534"	74° 37' 446"	4908	0-40
**3**	BC-03	12° 52' 509"	74° 45' 009"	5094	0-45
**4**	BC-04	12° 56' 216"	74° 41' 217"	5152	0-40
**5**	BC-05	13° 00' 003"	74° 37' 494"	5055	0-35
**6**	BC-06	13° 00' 014"	74° 44' 998"	4900	0-35
**7**	BC-07R	13° 29' 999"	74° 29' 982"	5227	0-40
**8**	BC-08R	15° 59' 993"	75° 29' 981"	5150	0-40
**9**	BC-09	15° 00' 011"	75° 29' 998"	4856	0-40
**10**	BC-10	13° 37' 498"	75° 29' 990"	5280	0-40
**11**	BC-11R	13° 37' 497"	75° 37' 494"	5252	0-30
**12**	BC-12	13° 29' 995"	75° 37' 493"	5180	0-35
**13**	BC-13	13° 33' 743"	75° 33' 743"	5187	0-40

Supplementary Document

The evolutionary history was inferred using the Neighbor-Joining method. The optimal tree with the sum of branch length = 2.76677748 is shown. The percentage of replicate trees in which the associated taxa clustered together in the bootstrap test (100 replicates) is shown next to the branches. The tree is drawn to scale, with branch lengths in the same units as those of the evolutionary distances used to infer the phylogenetic tree. The evolutionary distances were computed using the Maximum Composite Likelihood method and are in the units of the number of base substitutions per site. The rate variation among sites was modeled with a gamma distribution (shape parameter = 1). The differences in the composition bias among sequences were considered in evolutionary comparisons. The analysis involved 110 nucleotide sequences. All ambiguous positions were removed for each sequence pair. There were a total of 1803 positions in the final dataset
